# Pharmacological and mechanical properties of isolated pig coronary veins

**DOI:** 10.3389/fphys.2023.1275736

**Published:** 2023-11-02

**Authors:** Bowen Wang, Zhi Qin, Mei Li, Anders Arner, Stig Steen

**Affiliations:** ^1^ Department of Clinical Sciences, Lund University, Lund, Sweden; ^2^ Igelösa Life Science AB, Lund, Sweden

**Keywords:** coronary vein, coronary artery, length-tension relationship, relaxation responses, xenotransplantation

## Abstract

Recent successful cardiac transplantation from pig to non-human primates and the first pig-to-human transplantation has put the focus on the properties of the pig heart. In contrast to the coronary arteries, the coronary veins are less well characterized and the aim was to examine the mechanical and pharmacological properties of coronary veins in comparison to the arteries. Vessel segments from the left anterior descending coronary artery (LAD) and the concomitant vein were isolated from pig hearts in cardioplegia and examined *in vitro*. The wall thickness, active tension and active stress at optimal circumference were lower in coronary veins, reflecting the lower intravascular pressure *in vivo*. Reverse transcription polymerase chain reaction (RT-PCR) analysis of myosin isoforms showed that the vein could be characterized as having a slower smooth muscle phenotype compared to the artery. Both vessel types contracted in response to the thromboxane agonist U46619 with EC_50_ values of about 20 nM. The artery contracted in response to acetylcholine. Precontracted arteries relaxed in noradrenaline and substance P. In contrast, the veins relaxed in acetylcholine, contracted in noradrenaline and were unresponsive to substance P. In conclusion, these results demonstrate significant differences between the coronary artery and vein in the smooth muscle properties and in the responses to sympathetic and parasympathetic stimuli.

## 1 Introduction

Extensive efforts are currently invested in increasing the number of hearts available for transplantation, to solve the problem with shortage of suitable organ donors. Pig-to-human xenotransplantation is a promising venue, which has been advanced by recent successful pig-to-non-human primate transplantation (Langin et al., 2018; Reichart et al., 2020). A first transplantation from pig to human was performed early in 2022 using a heart from a donor pig with genes edited to prevent rejection (Reardon, 2022; Sade and Mukherjee, 2022). Although this approach is still under development, it has put a focus on the need for further understanding of the properties of the pig heart and its coronary circulation.

Previous work on the coronary artery of the pig has characterized agonist responses and demonstrated substance P- induced endothelial relaxation [e.g., (Qin et al., 2020; Arlock et al., 2023)]. In contrast, very little is known regarding the coronary veins. Even if the veins only contribute with a minor fraction (<10%, in a cat model) of the coronary flow resistance under control conditions, their relative contribution to resistance can increase to about 30% during arterial dilatation (Chilian et al., 1989), suggesting that venous tone is not negligible. Studies in the dog heart have shown that the venous pressure can also affect the ventricular wall diastolic distensibility (Watanabe et al., 1990), which further stresses the physiological role of venous tone. Previous studies in the guinea pig have shown that the veins are extensively innervated and can contract and relax in response to different agonists (Gulbenkian et al., 1990; Gulbenkian et al., 1994). However, information on the mechanical properties and main agonist responses of the coronary veins, in particular from larger animals (the pig) is not available.

The aim of this study was to investigate the mechanical and pharmacological properties of the cardiac veins in pigs. The vessels were obtained from cardioplegic pig hearts under similar conditions as during cardiac transplantation and compared to concomitant coronary arteries using an *in vitro* myograph system. We report on the smooth muscle phenotype, mechanical properties and on the contractile and relaxant responses to transmitters/hormones of the sympathetic and parasympathetic systems in these vessels.

## 2 Materials and methods

### 2.1 Animals and preparations

Twenty-nine healthy Swedish Landrace pigs of both sexes (20 males, all castrated, and 9 females) with a weight in the range of 30–55 kg (72–130 days old) were used. The animals were kept at the facility for approximately 1 week before the start of the experiments. All animals received care in compliance with the European Convention for the Protection of Vertebrate Animals Used for Experimental and Other Scientific Purposes (Directive 2010/63/EU). The Ethics Committee of the University of Lund approved the study (No. 5.8.18.15906-2020).

The animals were sedated with ketamine 750 mg (Ketaminol Vet. Intervet, Boxmeer, Netherlands), xylazine 100 mg (Rompun Vet, Bayer, Kiel, Germany), and atropine 0.5 mg (Atropine, Mylan AB, Stockholm, Sweden). Fentanyl 4 μg/kg body weight (Fentanyl B.Braun, Melsungen, Germany) and midazolam 0.4 mg/kg (Midazolam Panpharma, Panpharma S.A., Trittau, Germany) were given through an ear vein catheter. Midazolam 25 mg and rocuronium 20 mg (Rocuronium Fresenius Kabi, Bad Homburg, Germany) were given intravenously, then endotracheal intubation was performed, and general anesthesia was maintained with a continuous intravenous infusion of ketamine (200 mg/h), midazolam (6 mg/h) and rocuronium (40 mg/h). Ventilation with a minute volume of 100–150 mL/kg and a frequency of 20 breaths/minute was used. Heparin 500 IU/kg body weight (Heparin, LEO Pharma, Malmö, Sweden) was given intravenously.

A median sternotomy was performed, and the heart was exposed. The pericardium was opened, and a cardioplegic cannula was placed in the ascending aorta. After clamping the aorta, cardiac arrest was induced with St. Thomas cardioplegic solution (Plegisol, Pfizer, Sollentuna, Sweden, modified with increased KCl to 23 mM). The heart was rapidly excised and placed in 4 °C cardioplegic solution and allowed to cool down for at least 0.5 h at 4 °C. The heart was transferred to a dissecting dish containing 4 °C Krebs’ solution and pinned down to a silicone bottom. The Krebs’ solution had the following composition (in mM): NaCl 123, NaHCO_3_ 20, KH_2_PO_4_ 1.2, KCl 4.7, MgCl_2_ 1.2, CaCl_2_ 1.5, glucose 5.5. In experiments using noradrenaline, 0.03 mM EDTA was added for complexing trace amounts of metal ions that might accelerate oxidation of the agonist. Left anterior descending coronary arteries (LAD) and coronary veins (adjacent to the LAD) were dissected, freed from adipose and connective tissue and cut into rings 2–3 mm in length under microscope.

### 2.2 Circumference-tension relationships

The ring segments of the coronary vessels were suspended between two stainless-steel pins (0.2 mm in diameter) in 5 mL organ baths containing Krebs’ solution. The solution was equilibrated with 95% oxygen (O_2_) and 5% carbon dioxide (CO_2_) to give a pH of 7.4 at 37°C. One pin was attached to a Grass FT03 transducer, and the other was fixed to an adjustable micrometer screw for length adjustment. The isometric force signal was recorded on a computer by using a digital recording system (ADInstruments Ltd., Oxford, United Kingdom). The vessel rings were pre-stretched to a low passive force, approximately 2 mN, and allowed to equilibrate for at least 30 min. Contractions were induced by adding depolarizing high-K^+^ solution (80 mM KCl added to the Krebs’ solution) to examine the viability of the vessels. After the contraction had reached a stable plateau, the vessels were relaxed by changing to the normal Krebs’ solution. We chose activation with high-K^+^ solution since this mode of activation is reversible and KCl can be fully washed out. We have no evidence that subsequent contractions or relaxations are influenced by the high-K^+^-contractions. After three initial contractions, the circumference of segments was reduced to give zero passive force. Starting from the completely unloaded state, the circumference was increased in a stepwise manner by manual adjustment of the distance between the pins. After each step, the vessels were activated by high-K^+^ solution for 5 min to reach a plateau tension and then relaxed for 10 min. The stepwise increase of circumference was terminated when the active force was lower than that at the previous step. Since the passive length-tension relationship is very non-linear, the stretches were imposed to reach stepwise increases in passive tension. The length was recorded after each step. Active force was measured at the plateau of contractions. Passive force was measured in the relaxed state immediately before the high-K^+^ activation. The distance between the inner edges of the two pins was recorded using a microscope after each step and the internal circumference was calculated. Wall tension was calculated as force divided by 2 times the segment length. Optimal circumference was defined as the inner circumference where the vessel reached the maximum active tension. The wet weight of the vessel segments was measured after the experiments. Wall thickness was estimated as weight/(ρ × inner circumference × segment length), where ρ (density) was assumed to be 1.06 mg/mm^3^. This value was presented by Murphy (Murphy et al., 1980) and we expect that the values for the coronary vessels are similar. Wall stress was computed as wall tension divided by wall thickness. The equivalent pressure at optimal circumference was estimated using the Laplace equation: pressure = (active + passive tension)/(internal circumference/2 π). We also estimated the pressure range sustained by the vessels assuming a thick-wall model (Lamé equations).

### 2.3 Determination of the sensitivity to contractile agonists

In a separate series of experiments, the sensitivity of the coronary vessels to contractile agonists was examined. The coronary artery and vein segments were mounted as described above and stretched to approximately 80% of the optimal circumference. Then the vessel segments were activated with high-K^+^ solution and relaxed with the Krebs’ solution 3 times. Force of the third initial contraction was used for normalization of subsequent force responses. The contractile agonists were noradrenaline (10^−8^-10^−5^ M), acetylcholine (10^−9^-10^−6^ M) and the thromboxane A2 analogue U46619 (10^−9^-10^−6^ M). Experiments with noradrenaline were also performed in the presence of propranolol (10^−6^ M). In a separate set of experiments, we examined the effects of phenylephrine (10^−7^-10^−5^ M) in the coronary artery. Concentration-response relationships were determined by adding the agonist cumulatively. The pharmacological compounds were obtained from Tocris (Bio-Techne, Abingdon, United Kingdom).

### 2.4 Responses in precontracted vessels

The coronary vessels segments were mounted and activated with high-K^+^ solution. After the initial responses were recorded, a contraction was induced with 10^−7^ M U46619 giving about 80% of the maximal U46619 response. When a stable plateau was reached, increasing concentrations of acetylcholine (10^−9^-10^−5^ M), noradrenaline (10^−9^-10^−5^ M), or substance P (10^−9^-10^−7^ M) were added cumulatively. The relaxant responses to the different concentrations of agonists were expressed in percentage of the U46619 induced pre-contraction. The endothelium-independent vasodilator papaverine (10^−7^ M) or Ca^2+^-free Krebs’ solution with 3 mM EGTA were added at the end of the experiment to determine the maximal relaxation response.

### 2.5 Determination of myosin isoform expression

RT-PCR analysis was used to analyze the content of mRNA for the two splicing variants of the smooth muscle myosin essential light chain [LC17a and LC17b, (Lofgren et al., 2002)]. Samples of the coronary artery and vein were isolated and stored in RNALater (Thermo Fisher). Samples of the aorta and urinary bladder smooth muscle were analyzed in parallel. RNA was extracted (Promega MiniPrep), converted to cDNA (Applied Biosystems, High-capacity reverse transcription kit) and subjected to PCR using primers for the 17 kDa myosin essential light chain (Forward: ATG​TGT​GAC​TTC​ACC​GAG​GAC; Reverse: CAT​TCA​GCA​CCA​TGC​AGA​C) amplifying the region with the insertion present in the LC17b form (GeneID: 396807). PCR using primers for beta-actin (Forward: GAC​TCA​GAT​CAT​GTT​CGA​GAC​CTT; Reverse: CAT​GAC​AAT​GCC​AGT​GGT​GC) was performed in parallel on the samples. The products were separated on 3.5% agarose gels stained with GelRed (Sigma Chemical Co.) and analyzed using an imaging system (Chemidoc XRS, BioRad). Two bands at 451 and 496 bp reflect the LC17a and LC17b, respectively. Their ratio of LC17b/total LC17 was determined from the intensity of the bands.

### 2.6 Statistical analysis

Results are expressed as mean ± standard error of the mean (SEM). Values of tension, stress, and pressure were calculated as described above. Statistical comparisons and curve fitting were performed using routines implemented in SigmaPlot14 (Alfasoft AB, Gothenburg, Sweden). Student’s t-test was used when comparing two groups and analysis of variance (ANOVA) when several groups were compared. For values not normally distributed, a non-parametric test (Mann-Whitney rank sum test) was used. N is the number of vessels; the number of animals is given in the text.

## 3 Results

### 3.1 Circumference-tension relationships

**FIGURE 1:**
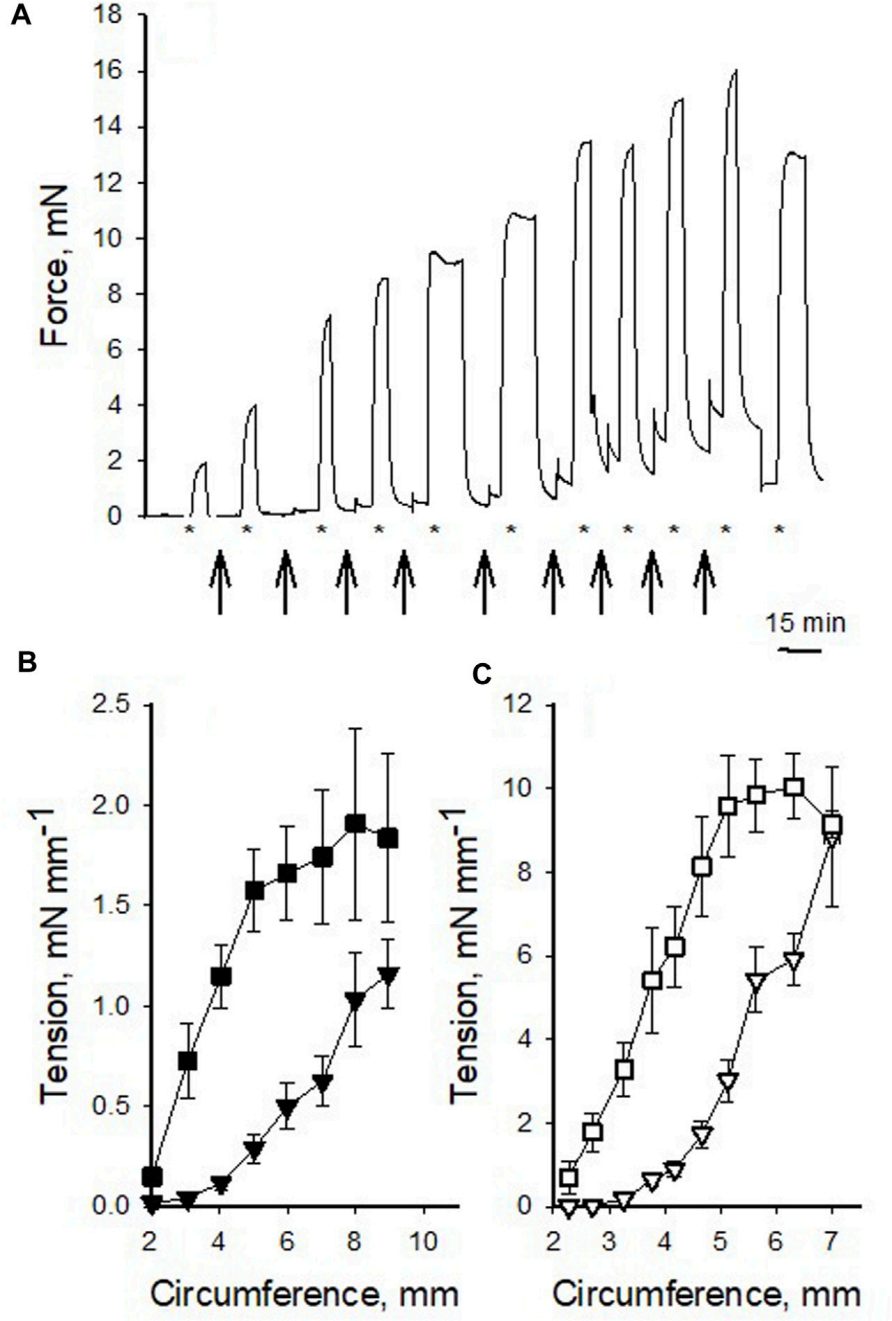
Circumference-tension relationships of pig coronary veins and arteries. **(A)** shows an original recording of force in a venous preparation repeatedly stretched (arrows) and activated with high K^+^ (indicated with * below the recording). Final contraction was elicited after returning to optimal stretch. **(B,C)** show summarized data of passive (triangles) and active (squares) tension at different circumference in veins (filled symbols) and arteries (open symbols), respectively. The data were grouped according to circumference values, *n* = 2–25 in each point, from 14 to 16 vessels from 5 to 6 animals, in veins and arteries, respectively. Note difference in scaling of *Y*-axes between **(B,C)**.

In a first series of experiments, we analyzed the mechanical properties of the pig coronary veins in comparison to the arteries. Figure 1A shows an original recording of force in an isolated vein preparation. The vessel was repeatedly stretched and activated with high K^+^ at each circumference and Figures 1B, C show the summarized data, for both vessel types. In the arteries (Figure 1C), the passive tension increased in an exponential manner with stretch and the active tension showed a bell-shaped relationship, where the maximal active tension was observed at a circumference of about 6 mm. The veins (Figure 1B) showed similar characteristics, albeit with significantly lower active and passive tension at the optimal circumference, which was about 8 mm. Table 1 summarizes the mechanical and structural properties of the two coronary vessel types. Both passive and active tension at optimal circumference were significantly lower in the veins. Wall thickness was estimated from vessel dimensions and weight. The veins had significantly smaller wall thickness. The calculated active stress (i.e., force per wall cross-sectional area) was also lower. Using the law of Laplace for thin-walled cylinders together with the mechanical and structural data, we estimated the maximal active pressure sustained by active and passive tension at optimal circumference. The estimated pressures were 143 and 20 mmHg in arteries and veins respectively, reflecting the difference in pressure levels sustained by these vessels *in vivo*. Since the wall thickness of the artery was about 12% of the diameter, the thin-wall model might not be applicable. We therefore estimated the pressures using Lamé equations for thick-walled cylinders. In this model, wall stress varies throughout the wall, and the sustained pressures would be different depending on where the total stress is located. However, using this model, we also found similar difference in the sustained pressure (arteries: 122–160 mmHg and veins: 18–22 mmHg).

**TABLE 1:** Mechanical and structural characteristics of pig coronary veins and arteries.

	Vein	Artery	Significance
Optimal circumference (Co), mm	7.63 ± 0.52, *n* = 14	5.92 ± 0.22, *n* = 16	*p* < 0.01
Active tension at Co, mN mm^−1^	2.21 ± 0.38, *n* = 14	11.8 ± 1.0, *n* = 16	*p* < 0.001
Passive tension at Co, mN mm^−1^	1.03 ± 0.18, *n* = 14	6.16 ± 0.65, *n* = 16	*p* < 0.001
Active stress at Co, mN mm^−2^	12.9 ± 2.0, *n* = 14	46.2 ± 4.1, *n* = 16	*p* < 0.001
Wall thickness, mm	0.17 ± 0.01, *n* = 14	0.26 ± 0.02, *n* = 16	*p* < 0.001

Optimal circumference (Co) for active tension and the active and passive tension at Co are shown. Wall thickness was determined from vessel dimensions and wet weight. Active stress (i.e., force per cross/sectional area) was calculated from wall thickness and tension. Five and 6 animals were used for veins and arteries, respectively. Statistical comparisons were made using Student’s t-test and Mann-Whitney rank sum test for active tension and stress.

### 3.2 Contractile responses to pharmacological agonists

**FIGURE 2:**
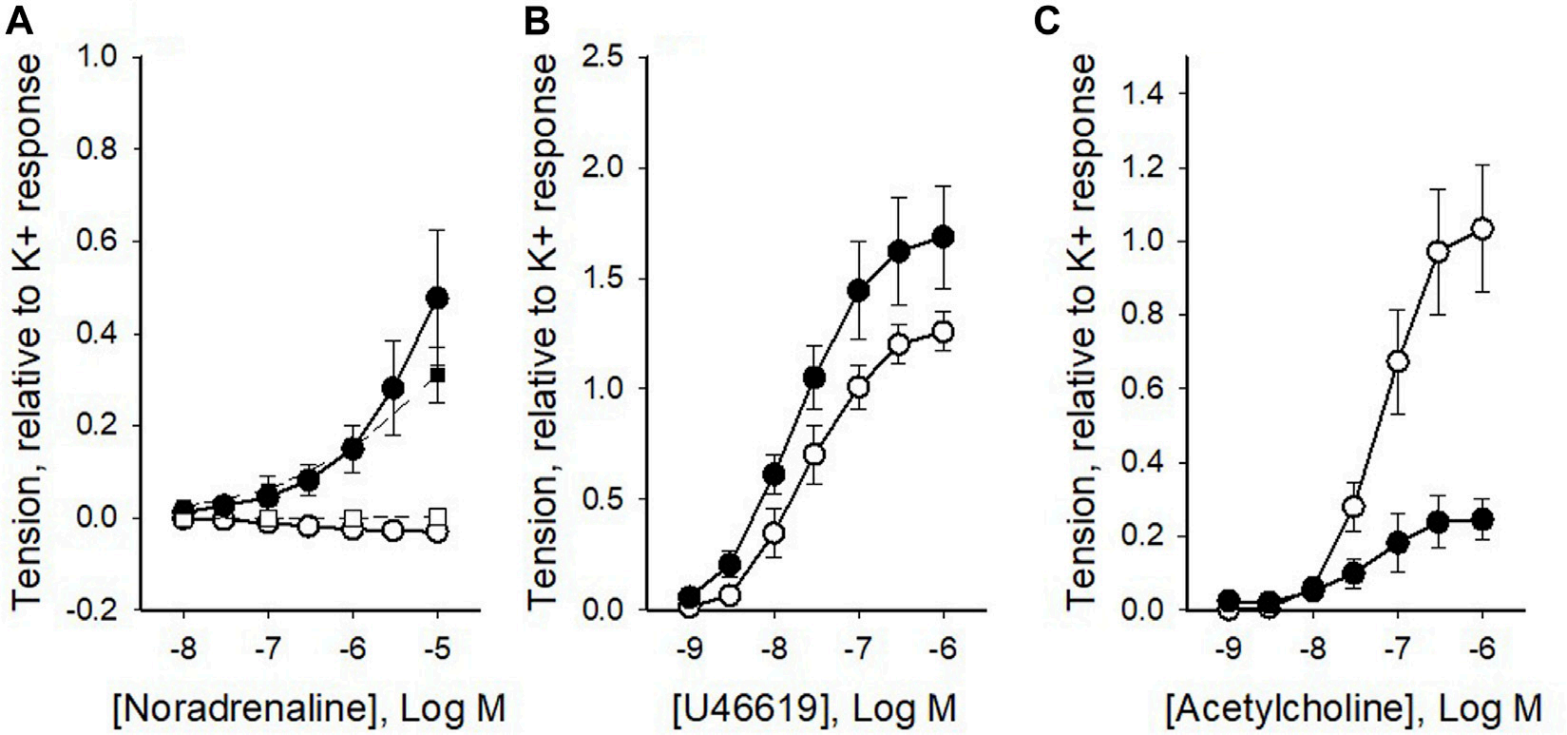
Summarized data of the contractile responses to noradrenaline **(A)**, U46619 **(B)** and acetylcholine **(C)** from pig coronary veins (filled symbols) and arteries (open symbols), respectively. Squares in **(A)** show noradrenaline responses in the presence of 10^−6^ M propranolol. Tension, relative to high-K^+^ tension, is shown against concentration of the respective agonist. The mean values of each relationship in panels b and c were fitted to *Eq*. *1*. N = 6-14, from 2-6 different animals.

Our next step was to compare the sensitivity to contractile agonists in the two vessel types. We observed that the coronary arteries did not show a significant response to adrenergic activation with noradrenaline, whereas coronary veins showed significant contraction in response to high concentrations of noradrenaline (Figure 2A). The lack of contraction in noradrenaline was also noted in the presence of the beta-blocker propranolol (10^−6^ M, squares in Figure 2A; data did not differ from data in the absence of blocker). In a separate set of experiments, we demonstrated that the alpha_1_ agonist phenylephrine did not contract the artery. Figure 2B shows the active tension at different concentrations of the thromboxane agonist U46619. The veins gave higher relative tension at saturating concentrations of the agonist. A hyperbolic Hill equation (*Eq*. *1*), in the form T = Max*c^*h*^/(c^*h*^ + *EC*_*50*_^*h*^), was fitted to the tension (T) and concentration (c) data, to determine the maximal response (*Max*), the concentration giving half maximal tension (*EC*_*50*_) and the steepness Hill coefficient (*h*). The mean values of curve fits to individual vessels are shown in Table 2. The veins had significantly higher maximal tension at saturating U46619 concentration compared to the arteries, and a slightly lower *h*-value and similar *EC*_*50*_. Figure 2C shows the relationship for the muscarinic agonist, acetylcholine, which gave a significantly more prominent activation in the coronary arteries compared to the veins. The corresponding data from the curve fits to *Eq*. *1* (Table 2) show that the maximal tension at saturating concentration was significantly higher in the arteries. Since the responses to acetylcholine were so small in the veins, we could not perform curve fits to determine the *h* or *EC*_*50*_ parameters.

**TABLE 2:** Pharmacological reactivity of pig coronary veins and arteries.

	Vein	Artery	Significance
Noradrenaline			
Maximal response, relative to high-K^+^ tension	0.48 ± 0.15, *n* = 14	−0.03 ± 0.01, *n* = 12	*p* < 0.001
U46619			
Maximal response, relative to high- K^+^ tension	1.68 ± 0.24, *n* = 8	1.26 ± 0.09, *n* = 14	*p* < 0.05
Hill (*h*) coefficient	1.09 ± 0.07, *n* = 8	1.70 ± 0.15, *n* = 16	*p* < 0.01
*EC*_*50*_, log M	−7.72 ± 0.04, *n* = 8	−7.56 ± 0.09, *n* = 16	n.s.
Acetylcholine			
Maximal response, relative to high-K^+^ tension	0.25 ± 0.06, *n* = 8	1.03 ± 0.17, *n* = 14	*p* < 0.01
Hill (*h*) coefficient		1.46 ± 0.08, *n* = 15	
*EC*_*50*_, log M		−7.02 ± 0.08, *n* = 15	

Sensitivity to noradrenaline, the thromboxane analogue U46619 and acetylcholine were determined, and the hyperbolic Hill equation *Eq*. *1* was fitted to the data to determine the maximal response at saturating concentration, the Hill (*h*) coefficient and the *EC*_*50*_ values. The maximal response is given relative to the high-K^+^ induced tension. Three to six animals were used for arteries and veins, respectively. Statistical comparisons were made using Student’s t-test or Mann-Whitney rank sum test for the maximal response to noradrenaline, U46619, and acetylcholine (in the veins for acetylcholine curve fits could not be performed and the maximal response was obtained from tension at the highest dose).

### 3.3 Responses in precontracted vessels

**FIGURE 3:**
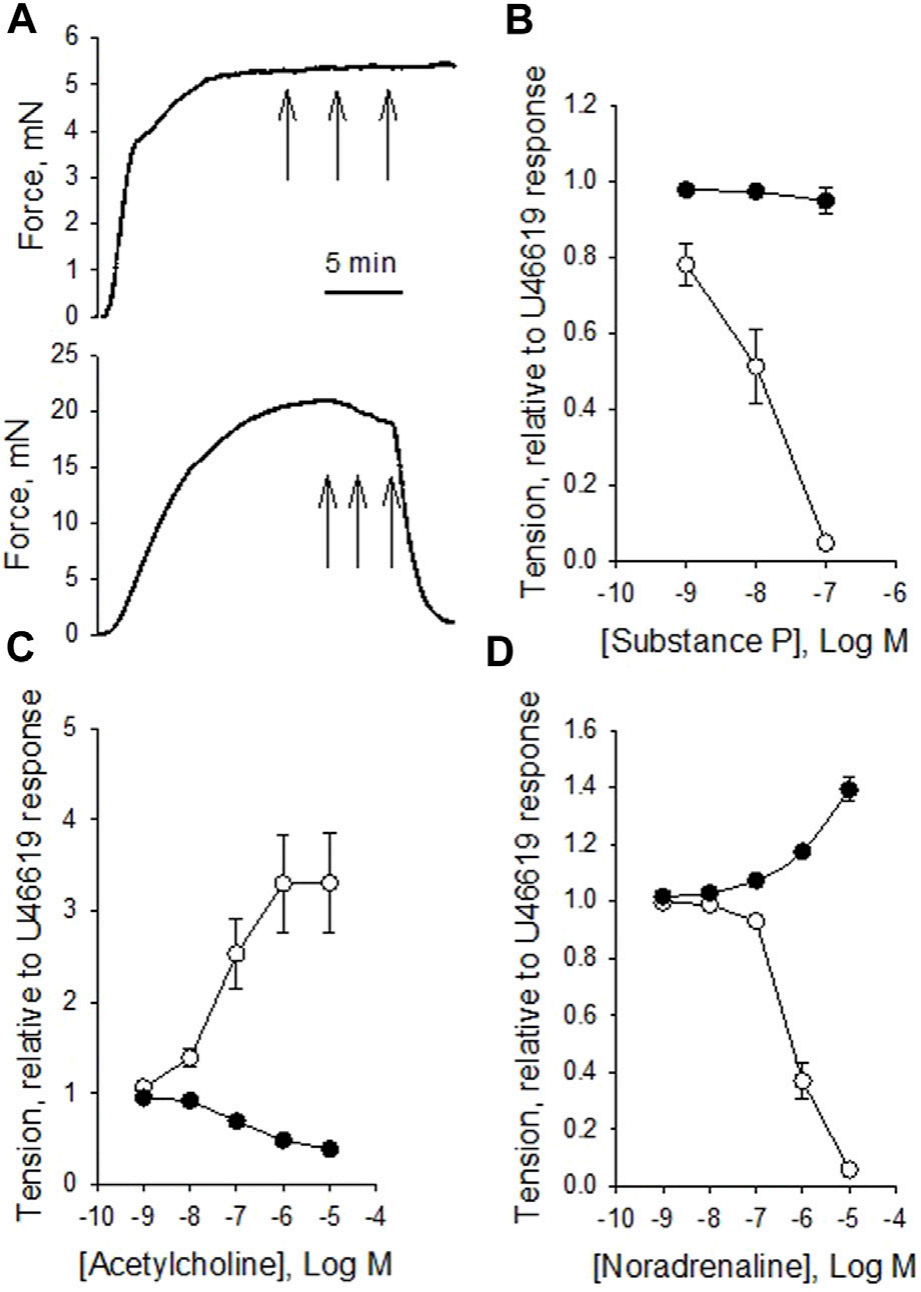
Original force recordings of a vein [**(A)** upper trace] and an artery [**(A)** lower trace] first activated with 10^−7^ M U46619 and exposed to different concentrations of substance P (10^−9^, 10^−8^ and 10^−7^ M, indicated by arrows). Summarized data of the responses to substance P, acetylcholine, and noradrenaline in precontracted vessels are shown in **(B–D)**, respectively. Tension is related to the precontraction in U46619. N = 12-15, from 3-4 animals for arteries (open symbols) and veins (filled symbols), respectively.

To explore relaxant responses, we precontracted the vessels with 10^−7^ M U46619, and then introduced substance P, acetylcholine or noradrenaline. Figure 3A shows original traces of force responses to substance P in a vein (upper trace) and artery (lower trace). Substance P relaxed the arteries but had almost no effect in veins (Figure 3B). Substance P also relaxed coronary arteries precontracted with Acetylcholine (10^−6^ M), relaxation to 16% ± 4%, n = 6 of the acetylcholine-induced force suggesting intact endothelial function also under these conditions. Acetylcholine induced a prominent contraction in the artery on top of the U46619 force (Figure 3C) consistent with the results from non-precontracted vessels (cf. Figure 2C). In contrast, the veins reacted with a clear relaxation in response to acetylcholine. Noradrenaline induced significant coronary artery relaxation of U46619 precontraction, whereas the coronary veins contracted (Figure 3D). The relaxation of the coronary artery in response to noradrenaline could be reversed by addition of propranolol (10^−6^ M), showing that the relaxation is due to beta receptor activation. Papaverine 10^−7^ M induced an almost full relaxation, stabilizing at values below about 6% of the U46619 force value for veins and arteries. A subsequent change to Ca^2+^-free EGTA solution gave a relaxation to 2%. Table 3 summarizes the responses to substance P, acetylcholine and noradrenaline in the precontracted vessels.

**TABLE 3:** Responses to pharmacological agonists in precontracted coronary arteries and veins.

	Vein	Artery	Significance
Substance P			
Tension at max. dose, relative to *U46619* tension	0.95 ± 0.03, *n* = 13	0.05 ± 0.01, *n* = 15	*p* < 0.001
Acetylcholine			
Tension at max. dose, relative to *U46619* tension	0.39 ± 0.07, *n* = 12	3.31 ± 0.54, *n* = 12	*p* < 0.001
Noradrenaline			
Tension at max. dose, relative to *U46619* tension	1.39 ± 0.04, *n* = 12	0.06 ± 0.01, *n* = 12	*p* < 0.001

The responses to substance P, acetylcholine and noradrenaline in vessels precontracted with 10^−7^ M U46619 were determined. The tensions at maximal dose of the agonists are given relative to the U46619 induced tension. Three to four animals were used for arteries and veins, respectively. Statistical comparisons were made using Student’s t-test or Mann-Whitney rank sum test.

### 3.4 Expression of essential smooth muscle myosin light chain isoforms

**FIGURE 4:**
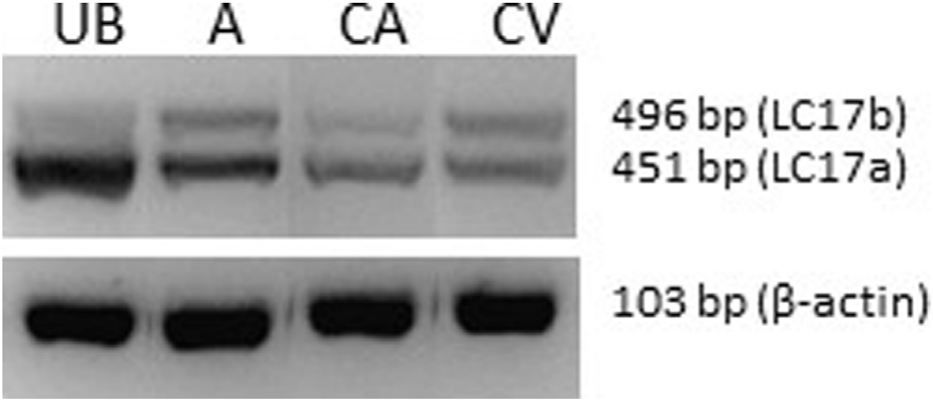
Photograph of an agarose gel with separation of PCR products (LC17a/b) from urinary bladder (UB), aorta (A), coronary artery (CA) and coronary vein (CV). The lower photograph shows beta-actin PCR products from the same samples.

We used the expression pattern of the 17 kDa essential smooth muscle myosin light chain as an indicator of the smooth muscle phenotype. Smooth muscle with slow contractile kinetics and adaptation for more sustained glucose and lipid metabolism has been associated with a higher expression of the larger LC17b isoform (Malmqvist and Arner, 1991; Boberg et al., 2018). Figure 4 shows gel separation of PCR products from coronary artery and vein, and for comparison, the urinary bladder and aorta. As seen in the picture, the urinary bladder has mainly the fast isoform LC17a (LC17b/LC17: 7% ± 3%, n = 6), and the aorta has a higher expression of LC17b (LC17b/LC17: 40% ± 7%, n = 5), reflecting a slow phenotype compared to the bladder (*p* < 0.001, ANOVA). Values from both coronary vessels were significantly different (*p* < 0.001) from the urinary bladder, suggesting that the vessels are of a comparatively slow phenotype. The coronary artery had an LC17b/LC17 ratio of 31% ± 9%, n = 7 and the coronary vein a significantly (*p* < 0.01) higher LC17b/LC17 ratio of 45% ± 5%, n = 7 suggesting that the vein was of a slower phenotype compared to the artery.

## 4 Discussion

We present a first detailed analysis of the mechanical and pharmacological properties of pig coronary veins, in comparison to coronary arteries. The pig coronary arteries have previously been examined in studies on, e.g., vascular aging (Murohara et al., 1991), transplantation (Arlock et al., 2023), endothelial function (Matsumoto et al., 1993) and exercise (Parker et al., 1994), whereas the properties of the veins are poorly understood. The two examined vessels in the present study (left anterior descending coronary artery and the adjacent coronary vein) are located on the surface of the heart in the anterior interventricular sulcus. Not surprisingly, the veins had thinner walls and had less active and passive wall tensions at optimal stretch, which would reflect the lower intravascular pressures sustained *in vivo*, compared to the arteries. The lower active tension in the veins was associated with a lower active stress (i.e., active force generated per wall cross-sectional area). Smooth muscle exhibits a significant heterogeneity in contractile properties, which is also reflected in expression of metabolic and signaling proteins (Malmqvist and Arner, 1991; Boberg et al., 2018). The essential smooth muscle myosin light chain (LC17) has been shown to be a good marker for the phenotype, with slow smooth muscles (like the aorta) having more the of LC17b form compared to faster types (like the urinary bladder). We show here that both the coronary vessels are intermediate types, and that the vein could be identified as a slower type compared to the artery. It should be noted that the smooth muscle phenotypes are also reflected in their metabolic properties, e.g., in expression of pathways in glucose and lipid metabolism which might have an impact on their responses to pathological conditions.

We show that thromboxane activation gave significant contractions in the veins, a property previously described for coronary arteries (Ellis et al., 1976; Toda, 1984). In this aspect, arteries and veins reacted in a similar manner, demonstrating active contractile thromboxane receptors in both vessel types, suggesting that release of thromboxane A_2_, e.g., from platelet aggregation [cf. (Shepherd and Vanhoutte, 1986)] or monocytes/macrophages in graft rejection (Zhao et al., 1997) would contract both the arteries and veins.

We observe, however, significant differences in the pharmacological reactivity to adrenergic and cholinergic agonists between these two coronary vessels from the pig. It has previously been shown that pig coronary arteries with endothelium removed contract *in vitro* in response to alpha_1_ adrenergic agonists (Yan et al., 1998). However, *in vivo* administration of alpha receptor blockers to humans does not have major effects on coronary resistance (Hodgson et al., 1989), suggesting that adrenergic tone, at least at rest, is minimal. In contrast, unspecific beta blockers increased coronary resistance (Hodgson et al., 1989), suggesting a balance between the contractile alpha- and the relaxant beta-receptors. We report that noradrenaline, which has strong effects on alpha_1_ receptors, but also effects on beta, does not contract the pig coronary arteries *in vitro*, but instead, induces a significant relaxation in precontracted state. These results are consistent with reports from canine and porcine coronary arteries showing relaxant beta responses (Horst and Robinson, 1985; O'Donnell and Wanstall, 1984). We conclude that the lack of contraction in response to noradrenaline in the artery is due to lack of alpha_1_ receptor function, since both phenylephrine and noradrenaline in the presence of betablocker failed to induce contraction. Since our study was focused on comparisons between arteries and veins, we did not further examine the type of beta receptors mediating the noradrenaline-relaxant responses in the arteries, rather conclude that the net effect of the physiological agonist noradrenaline is relaxation of the coronary arteries in the pig. It should also be noted that our study, comparing arteries and veins of similar size, was performed on larger vessels and that adrenergic responses in smaller arterial vessels might differ.

We report that noradrenaline induced a significant contraction in the coronary veins. In larger pig coronary vessels (*sinus coronarius*) from slaughterhouse material, noradrenaline induced contractions that were significantly attenuated by alpha receptor blockers but unaffected by beta blockers (Bergner et al., 1988). Our data suggest that an important effect of sympathetic activation would be contraction of coronary veins and relaxation of coronary arteries. This would be physiologically relevant since increased sympathetic tone and increased heart work, would require dilatation of the arteries and increased flow. We can only speculate on the physiological significance of the venous contraction. It might be relevant to increase the venous resistance and capillary pressure in situations with increased heart rate, shorter diastolic time and increased ventricular wall tension, to keep the capillary vascular bed open for better perfusion or for balancing the hydrostatic pressures in the Starling equilibrium. Coronary flow is highest during diastole and decreases during systole when wall tension is high. However, the cardiac muscle contraction during systole promotes venous return to the right atrium. Venous contraction and increased venous wall stiffness induced by increasing the sympathetic tone might, thus, also be important for the venous return in the coronary circulation.

A key relaxant agonist in the coronary arteries of the pig is substance P (Budrikis et al., 1999), an undecapeptide primarily released from sensory nerves surrounding the coronary vessels (Gulbenkian et al., 1990; Gulbenkian et al., 1994), and possibly also endothelial cells (Milner et al., 1989). We confirm the relaxant effect in the arteries showing that the endothelium is functional, and report that substance P does not affect tension in precontracted coronary veins. The sensitivity to substance P (full relaxation at 10^−7^ M) observed in our study was lower than that reported by Oltman et al. (1995) (full relaxation at 10^−8^ M), using preactivation with endothelin, KCl and prostaglandin, but similar to a previous report using U46619 preactivation (Qin et al., 2020). Thus, substance P induces a prominent arterial relaxation, but the sensitivity is dependent on the mode of precontraction. In the veins, acetylcholine is active in inducing endothelium mediated relaxation whereas substance P is inactive. In contrast, it has been reported that epicardial coronary veins from the guinea pig, precontracted with PGF2α showed a small and variable relaxation in response to substance P (Gulbenkian et al., 1994). In our study, the pig coronary veins, stably precontracted with U46619 relaxed promptly to acetylcholine, as discussed below, which suggests that the muscarinic receptors are present and that the endothelium is functional. Our substance P data thus show that relaxant neurokinin 1 receptors are lacking in the veins. These data suggest that situations activating sensory nerves in the heart, e.g., ischemia (Fu and Longhurst, 2009) can via axon reflexes relax coronary arteries without strong effects on the veins.

Acetylcholine contracted both arteries and vein, although veins gave significantly lower responses, about 25% of the arterial response. The results regarding arteries are consistent with previous reports from rabbits, pigs and humans (Ito et al., 1979; Kalsner, 1985; Corr et al., 1991) showing muscarinic receptor activation of contraction in porcine coronary arteries, most likely via smooth muscle M3 receptors (Entzeroth et al., 1990). Contractile factors released from the endothelium have also been suggested to play a role (Kurahashi et al., 2003). The relaxant effect of acetylcholine via arterial endothelium has been found to be minimal also in human coronary arteries while substance P relaxes (Berkenboom et al., 1989). In contrast, relaxant responses to acetylcholine in human coronary arteries *in vivo* have been reported (Collins et al., 1993). In pig coronary veins, we show that acetylcholine induced a significant relaxation of precontracted vessels, suggesting that the parasympathetic activation would have a relaxant effect on venous tone. The *in vivo* effects of acetylcholine in veins, i.e., relaxation or contraction, would thus most likely depend on the level of active tone. In this context, the muscarinic antagonist atropine has been used to relieve coronary arterial spasm in some situations (Hung et al., 2000), and this compound would partly counteract cholinergic dilation in the veins. Our results demonstrate a clear difference in the pig heart between artery and vein in response to acetylcholine. These *in vitro* data would thus suggest that parasympathetic activation induces contraction in arteries and relaxation in veins.

In conclusion, our results show differential responses in coronary arteries and veins from the pig to adrenergic and cholinergic stimulation; noradrenaline primarily relaxes the arteries and contracts the veins, whereas acetylcholine contracts the arteries and relaxes the veins. The effects of acetylcholine on coronary arteries might be different in humans, where the cholinergic endothelial-dependent relaxation in arteries appears to be present, a factor of possible importance in xenotransplantation. Both sympathetic and parasympathetic innervation of coronary vessels have been shown to be rich (Goodwill et al., 2017), and nerve release of transmitters, together with catecholamines from adrenals, would be the main source of the activator compounds. Interestingly, a subpopulation of acetylcholine producing leucocytes has recently been demonstrated (Olofsson et al., 2016), which might provide a source of this agonist via the blood stream, possibly in situations with vascular injury or inflammation recruiting leucocytes in the vicinity of the vascular wall.

## Data Availability

The raw data supporting the conclusion of this article will be made available by the authors, without undue reservation.
